# Evaluation of two new antibodies for recognition of CldU in DNA fiber assay applications

**DOI:** 10.17912/micropub.biology.001485

**Published:** 2025-01-31

**Authors:** Anthony Widjaja, Julia M. Sidorova

**Affiliations:** 1 Center for Genomics and Systems Biology , New York University, New York, New York, United States; 2 Laboratory Medicine and Pathology, University of Washington, Seattle, Washington, United States

## Abstract

DNA fiber assays are indispensable tools for studying DNA damage and replication stress responses *in vivo* at the single replication fork level. These assays typically rely on antibodies recognizing IdU and CldU. Historically, the availability of CldU-reactive antibodies has been limited to one reagent (clone BU1/75(ICR1)). We validated two alternative antibodies for CldU detection in DNA fiber assays. One of these antibodies can be readily paired with a common IdU-reactive antibody, and we confirmed that it produces quantitatively similar CldU track length results vis-à-vis the BU1/75 antibody. The new reagents should boost versatility of DNA fiber assays, facilitating DNA replication research.

**Figure 1. Validation of CldU-reactive antibodies for DNA fiber assays:**
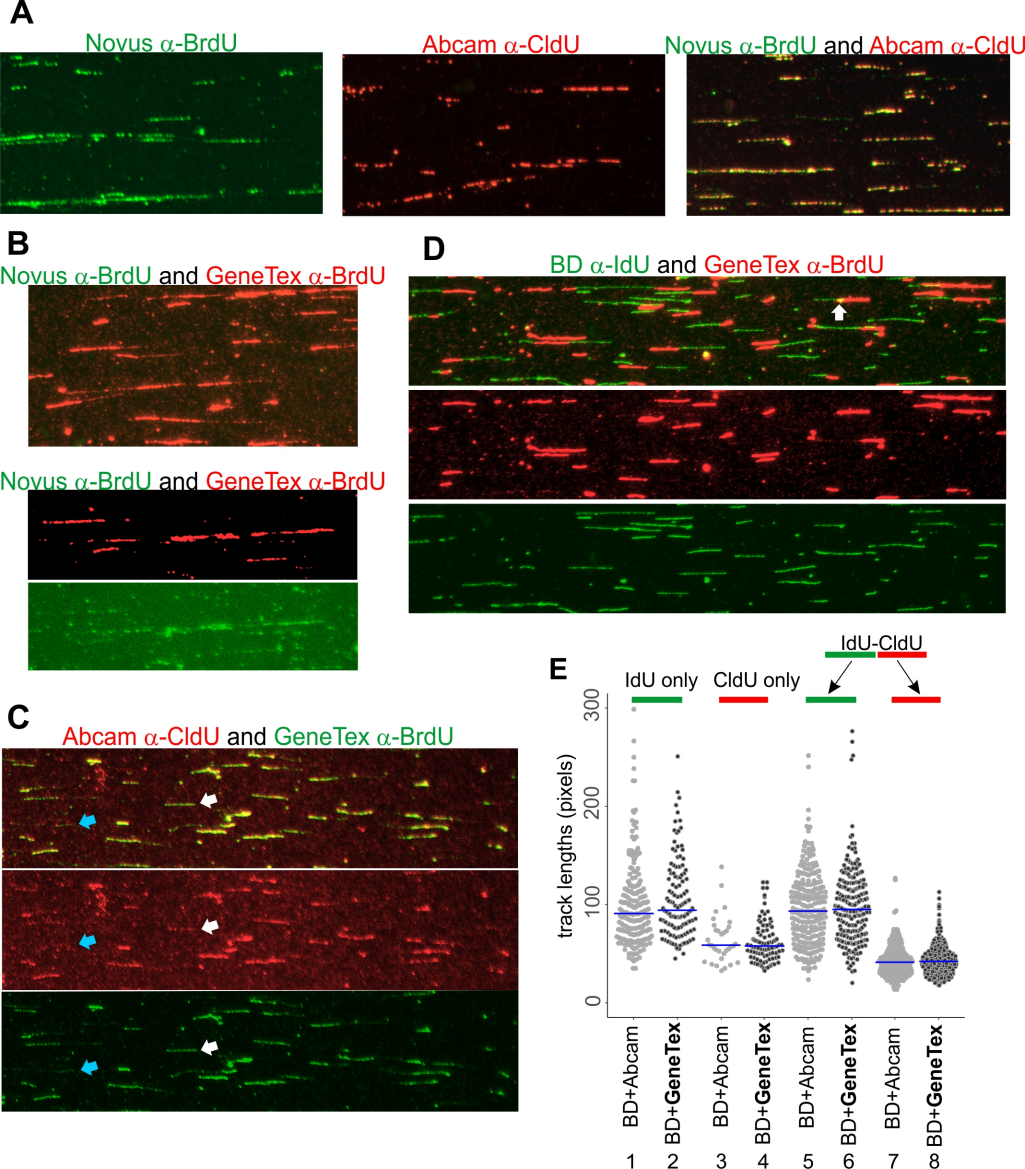
**A** ) Genomic DNA of A549 cells sequentially pulse-labeled with IdU for 40 min and then with CldU for 40 min, was stretched and stained with the indicated antibodies, used separately (left and center panels) or in combination (right panel). **B** ) The same sample as in (A) was stained with an indicated antibody combination. Top panel, a merged (red and green channels) image. Bottom panel, a split channel view a different field stained by the same antibody combination. **C–D** ) The same sample as in (A) was stained with the indicated antibody combinations. Shown are merged and single-channel views of the same fields. See text for explanations for blue and white arrows. **E** ) Genomic DNA from primary fibroblasts pulse-labeled sequentially with IdU for 40 min and then with CldU for 20 min was stretched, stained with the BD α-IdU antibody and either the Abcam or the GeneTex α-CldU antibodies, and IdU and CldU track lengths in IdU-only (lanes 1,2), CldU-only (lanes 3,4), and IdU-CldU (two-segment, lanes 5-8) tracks were quantified and plotted. For each track type, pairwise comparisons of the track length values measured in the slides stained with Abcam vs. GeneTex antibodies indicate no statistical difference between the value sets, with p values ≥ 0.3 in K-S tests.

## Description

DNA fiber assays are a group of techniques that display individual genomic DNA molecules stretched to near-contour length on glass support for the purposes of studying DNA replication and DNA damage response *in vivo* , and colocalization of replication with specific DNA sequences or with specific proteins (Chastain et al., 2006; Cohen et al., 2010; Datta & Brosh, 2022; Herrick & Bensimon, 1999; Jackson & Pombo, 1998; Norio & Schildkraut, 2001; Quinet, Carvajal-Maldonado, Lemacon, & Vindigni, 2017; Sidorova, Li, Schwartz, Folch, & Monnat, 2009). Introduced in the late nineties, DNA fiber assays have become a must-have tool for the labs that examine these processes in molecular detail. They are the only widely available, economical, and readily adoptable approach able to deliver quantitative information on how DNA replication and the associated events occurred inside a cell at the level of individual replication forks, although it should be noted that nanopore sequencing-based techniques are being developed to provide a similar level of resolution (Boemo, 2021; Hennion et al., 2020; Hennion, Theulot, Arbona, Audit, & Hyrien, 2022). The spread of DNA fiber assays has enabled the incredible progress of the last decade in the study of replication stress responses in human and animal cells, and the discovery of multiple pathways that support replication forks in normal cells and are altered in cancer, contributing both to chemosensitivity and to acquired chemoresistance (Berti et al., 2013; Berti, Cortez, & Lopes, 2020; Chaudhuri et al., 2016; Cong et al., 2021; Vindigni & Lopes, 2017).

For a typical DNA fiber assay, live cells are sequentially pulse-labeled with two different nucleoside analogs, CldU and IdU. Once DNA is stretched on glass and apurinized with hydrochloric acid, the analogs incorporated into DNA are stained using immunofluorescence, such that CldU and IdU can be visualized under the microscope as tracks (a.k.a. tracts) of different color. The two-color visualization allows to distinguish tracks left by ongoing forks versus terminated or other types of forks, as well as perturb replication for the duration of incorporation of only one label and then use the other label as an internal control for each individual fork. Historically, antibodies originally derived against BrdU are used for these purposes because these antibodies often exhibit reactivity to one of the other analogs, IdU or CldU. Reactivity to IdU appears more common, however, currently only one CldU-recognizing antibody is used in DNA fiber assay protocols (as exemplified by these recent methods publications (Biber & Wiesmüller, 2021; Halliwell, Gravells, & Bryant, 2020; Martins, Tirman, Quinet, & Menck, 2022; Moore, Jimenez Sainz, & Jensen, 2022)), and it is, at least in the US, now offered by only one vendor (Abcam, Cat. No. ab6326), to our knowledge.

Here we describe two more antibodies as recognizing CldU and suitable for use in DNA fiber assays. A mouse α-BrdU antibody offered by Novus (Cat. No. NBP2-44055) is listed as having cross-reactivity to CldU and to a lesser degree, to IdU. Its use in DNA fiber assays has not been tested. We used this antibody alone or together with the rat α-CldU on human A549 cell line samples that were sequentially pulse-labeled for 40 min with IdU and CldU. The Novus antibody produced a robust signal (Fig.1A, left panel), which completely overlapped with the signal produced by Abcam rat α-CldU ( Fig. 1A, right panel), suggesting that this antibody preferentially recognizes CldU under DNA fiber assay conditions.

A rabbit α-BrdU antibody offered by GeneTex (Cat. No. GTX128091) has no cross-reactivity information. We tested it in combination with the Novus antibody to determine if these two antibodies could be paired in DNA fiber assays to visualize, respectively, IdU and CldU. Unexpectedly, we observed no Novus-specific signal (Fig.1B, top panel), however, after a close examination this signal was found overlapping with and masked by the much brighter red fluorescence produced by the GeneTex antibody (Fig.1B, bottom panels). Further testing demonstrated that the GeneTex antibody signal overlaps with that of the Abcam α-CldU antibody when these two antibodies are used together on an IdU, then CldU-labeled sample (Fig.1C, top panel). A split-channel view of the images allows to appreciate the extent of the overlap (Fig.1C, middle and bottom panels). Single-channel images also reveal examples of tracks that are stained with intermediate intensity by the GeneTex antibody and with low intensity by the Abcam antibody (white arrows), or with low intensity by the GeneTex antibody and not by the Abcam antibody (blue arrows). The latter tracks in particular could indicate weak cross-reactivity of the GeneTex antibody to IdU. Alternatively, they and the tracks marked by the white arrows can represent DNA with low incorporation of label, which is more likely to go undetected in the red channel due to the overall weaker intensity and lower signal to noise ratio of the Abcam antibody staining in the experiment.

Nevertheless, the strong preference of the GeneTex antibody for CldU can be readily observed when this antibody is used together with the BD mouse antibody against IdU on the same sample. In this case, the GeneTex antibody recognizes non-overlapping tracks (Fig.1D, merged and split-channel views), except at some junctions between IdU and CldU labels, which is expected since both labels may be present in the cell for a short period of time after a switch from the first label pulse to the second (Fig.1D, white arrow). Importantly, single channel views demonstrate no reactivity to IdU by the GeneTex antibody despite a deliberately high exposure in the red channel. We conclude that this antibody is able to recognize CldU and can be used together with a mouse α-IdU antibody to reveal the dual-staining pattern standard to the DNA fiber assays.

To further characterize CldU recognition utility of the GeneTex antibody, we quantified track length distributions in a IdU, then CldU labeled sample stretched on two separate slides and stained with the BD antibody to IdU and either the Abcam or the GeneTex antibody to CldU. Lengths of IdU segments were measured as a control to verify that the two slides were not diversely affected by stretching and staining steps. As expected, IdU length values both in tracks that incorporated only IdU (terminated forks) and that sequentially incorporated IdU and CldU (ongoing forks), were similar (Fig.1E, lanes 1-2 and 5-6, p values respectively 0.311 and 0.61). Satisfyingly, CldU length values were also similar in ongoing forks as well as in CldU-only tracks (newly fired origins) between Abcam and GeneTex-stained slides, indicating that both antibodies produce quantitatively similar results for CldU track length distributions (Fig.1E, lanes 3-4 and 7-8, p values, respectively, 0.86 and 0.74). These data further validate the GeneTex α-BrdU antibody as an alternative to the Abcam antibody for identifying and measuring CldU tracks in DNA fibers.

In conclusion, our data indicate that two more CldU-reactive antibodies can be added to the DNA fiber analysis toolkit, increasing its versatility and decreasing dependence on a single antibody source for CldU recognition. Of these, the GeneTex antibody is a ready to go alternative to the Abcam antibody in DNA fiber assays as it can be paired with the α-IdU BD antibody commonly used in DNA fiber assays. The Novus antibody on the other hand, while able to recognize CldU in DNA fibers, will require further effort of finding a suitable IdU-recognizing partner antibody. Currently, all commercially available antibodies advertised as recognizing IdU, are produced either in mouse or in rat and to our knowledge none of the rat antibodies have yet been tested in DNA fiber assays. Nevertheless, the Novus antibody can certainly be used for single-label recognition, and in fact we have already used it in a combination of maRTA and FISH where this antibody is effective on formamide-denatured DNA (Lazarchuk et al., 2024) . We believe that our findings will be of help to the research community that relies on DNA fiber assays in their studies.

## Methods

Stocks of 5-iododeoxyuridine (IdU, Millipore-Sigma) was at 2.5mM in PBS, 5-chlorodeoxyuridine (CldU, Millipore-Sigma) was at 10mM in PBS. Genomic DNA isolates used in this study were from the A549 cell line and primary fibroblasts referenced, respectively, in (Lazarchuk, Nguyen, Brunon, Pavlova, & Sidorova, 2023) and (K. Kehrli et al., 2016). Cells were grown in high glucose Dulbecco Modified Minimal Essential Medium (DMEM) with L-glutamine, 10% fetal bovine serum, FBS, (Hyclone), and antibiotics in a humidified 5% CO2, 37°C incubator. Cells were seeded in 6-well plates, and after 1-2 days of growth to 50-70% confluence were sequentially labeled with 50μM IdU for 40 min and then 50μM CldU for 40 min (A549) or 20 min (primary fibroblasts). Cells were harvested after 20-30 min chase after removal of the CldU label and processed for microfluidics-assisted replication track analysis (maRTA) as described previously (Kehrli & Sidorova, 2014; Sidorova et al., 2009) . Working dilutions of Novus α-BrdU and GeneTex α-BrdU antibodies were, respectively, 1:300-500 and 1:200, and of Abcam and BD antibodies, respectively, 1:100 and 1:6 to 1:10. Incubation conditions for both of these antibodies were identical to those described in the above references for α-CldU and α-IdU staining. Microscopy of stretched DNAs was performed on the Zeiss Axiovert microscope with a 40x objective, and images were captured with the Zeiss AxioCam HRm camera. Track quantitation was performed using AxioVision software.

## Reagents

**BIOLOGICAL REAGENTS TABLE**

**Table:** 

REAGENT or RESOURCE	SOURCE	IDENTIFIER
Antibodies		
rat α-BrdU/CldU BU1/75 (ICR1)	Abcam Cat. No. ab6326	RRID:AB_305426
mouse α-BrdU/IdU	BD Biosciences Cat. No.347580	RRID:AB_10015219
mouse α-BrdU clone 29G6.E8	Novus Cat. No. NBP2-44055	RRID:AB_3307850
rabbit α-BrdU	GeneTex Cat. No. GTX128091	RRID:AB_11168976
goat anti-rabbit IgG (H+L) Alexa Fluor™ 488	Thermo Fisher Cat. No. A-11008	RRID:AB_143165
goat anti-mouse IgG (H+L) Alexa Fluor™ 488	Thermo Fisher Cat. No. A11001	RRID:AB_2534069
goat anti-rabbit IgG (H+L) Alexa Fluor™ 594	Thermo Fisher Cat. No. A-11012	RRID:AB_2534079
goat anti-rat IgG (H+L) Alexa Fluor™ 594	Thermo Fisher Cat. No. A-11007	RRID:AB_10561522
